# Application of Multifunctional Intelligent Suspension Treatment Beds in Nursing Care of Patients with Extensive Burns

**DOI:** 10.1155/2021/8922504

**Published:** 2021-12-16

**Authors:** Fangyan Chen, Liwen Xu, Guozhong Lv, Yugang Zhu, Jiang Chang, Fang Zou, Dan Sun

**Affiliations:** ^1^Department of Burns and Plastic Surgery, The Affiliated Hospital of Jiangnan University, Wuxi 214122, China; ^2^College of Nursing, Ningxia Medical University, Yinchuan City 750004, Ningxia Hui Autonomous Region, China

## Abstract

The nursing care of patients with extensive burns by using multifunctional intelligent suspension treatment beds was studied. 40 patients, including 30 males and 10 females, with extensive burns were nursed using multifunctional intelligent suspension treatment beds. First of all, the patients were given psychological care, which was patiently explained, so that they can overcome their fears and be treated with peace of mind. Second, the room temperature and bed temperature were closely monitored. Finally, special attention was paid to the adjustment of rehydration volume, regular detection of plasma electrolytes, prevention of electrolyte disorder, and dehydration. Besides, disinfection and isolation should be performed when using. The results showed that 4 cases (20%) were positive in group A and 8 cases (40%) were positive in group B on the 10th day after injury (*X*^2^=4.005), and the incidence of wound infection in group A was significantly lower than that in group B. The use of suspension beds in patients with extensive burns makes them safe and comfortable, and the whole body wound scabs healed faster, as well as the infection was minimized. A suspended bed is especially suitable for the clinical treatment of patients with extensive burns. The advantages of suspended bed can be fully realized by summarizing clinical experience.

## 1. Introduction

The development of society and the progress of science and technology have facilitated people's lives. On the other hand, they have led to frequent accidental injuries, and the number of burn patients has increased every year, which has caused great pain and injury and caused a heavy burden on society. According to the survey, there are about 10 million burn incidents every year. Suspended beds are widely used in Chinese burn centers for their advantages of quick drying, prevention and treatment of infection, relief of wound pain, and greatly reduced nursing workload [1]. A suspended bed is an air flow treatment unit consisting of a container filled with tiny silica sand, and the filtered air is sent to the silica sand through an air compressor to turn the silica sand into fluid. A buoyancy is formed by the flow of silica sand. Due to the buoyancy, the body can avoid pressure and keep the air flowing and relatively dry. At the same time, automatic water cooling, air cooling, and heat generation systems in the bed keep the bed temperature constant. In order to make the suspended bed fully perform its function and prevent infection, a special person should be made responsible for maintenance [2]. The Cleanliness of the air filter in the suspension bed and the condition of the water filter in the water channel should be regularly checked, and the filter element should be replaced in time, so as to avoid placing all kinds of sharp tools on the filter sheet. The disinfection sheet should be kept placed on the filter sheet, and the dirt should be cleaned and replaced in time. Disinfection of the suspension bed and the filter sheet has to be carried out regularly to prevent cross infection [3]. Many clinical diseases can induce hypernatremia, and hypernatremia can also occur in different stages of the same disease, which indicates that hypernatremia has a great influence on the occurrence and development of the disease and profoundly affects the treatment of the disease and the prognosis of patients. Hypernatremia will cause imbalance of osmotic pressure inside and outside cells, and a large amount of water and tissue fluid will enter the interstitial space, which will lead to dehydration and abnormal function of cells [4]. It has been pointed out that, after patients are complicated with hypernatremia, the brain cells will be seriously dehydrated, and the membrane potential of neuritis cells will also be affected, resulting in neurological dysfunction and abnormal brain functional areas, which will lead to aggravation of the disease and an increased risk of death. Patients with extensive burns will also undergo similar pathological changes, resulting in gradual aggravation of the disease and poor prognosis [5]. Hyperchloremia is also a kind of electrolyte disorder, and its causes are various, but pathological factors are the main ones [6]. It plays an important role in the regulation of various electrolytes in the kidney. Similarly, it plays a key role in the regulation of chloride ions through the action of various renal unit transporters. However, when the water loss exceeds the loss of sodium and chlorine, hyperchloremia will occur; however, when the kidney needs to treat excessive chlorine beyond its capacity, or when the exchange process between the chloride ion and bicarbonate is blocked, metabolic acidosis will be induced, which will lead to the increase of chloride ion content in the body and induce hyperchloremia [7]. Because of the diversity and complexity of the causes of hyperchloremia, how to treat this electrolyte disorder is largely determined by them. Reasonable adjustment of the infusion volume in the horizontal suspension bed is far more than that of the ordinary hospital bed, and it varies greatly with the burn area and the time after injury [8]. At present, there is not much information about this aspect. Alhazzani et al. put forward the formula of rehydration in the upper suspension bed of (2000 ml+(40.9) ± 37.43) ml, combined with the observation of urine volume per hour and peripheral circulation; it can basically satisfy the effective circulation, and there is no imbalance between water and electrolyte [9]. Trenta Coste et al. think that the mortality rate of patients with hypernatremia on the basis of the primary disease is as high as 69%, while the mortality rate of patients without hypernatremia is relatively low in the same period. The longer hypernatremia occurs in patients with severe craniocerebral injury, and the higher the absolute value of blood sodium, the higher the risk of death [10]. Loy and Obirieze think that patients with extensive burns complicated with hypernatremia and hyperchloremia in the early stage have their own characteristics, causes, and clinical manifestations, and their treatment is also special compared with other surgical diseases. Many factors may lead to hypernatremia and hyperchloremia. Patients with hypernatremia usually show typical nervous system symptoms. However, due to the existence of the primary disease, the hypernatremia-induced nervous system and related manifestations in many patients are often concealed and ignored [11]. Exploring the nursing method of using multifunctional intelligent suspension treatment beds to treat patients with extensive burns, it was found that using the advantages of suspension beds can minimize the pressure on the wound surface, thus reducing the risk of infection and scab dissolution. In this paper, 40 patients (30 males) with extensive burns were treated with multifunctional intelligent suspension treatment beds. 10 women were given psychological nursing, which was patiently explained, to overcome their fears and treat them with peace of mind. Room temperature and bed temperature were closely monitored. Special attention was paid to the adjustment of fluid infusion and regular checking of plasma electrolytes to prevent electrolyte disturbance and dehydration. Disinfection and isolation should be carried out well during the procedure. The results showed that 4 cases (20%) were positive in group A and 8 cases (40%) were positive in group B on the 10th day after injury. By the comparison between two groups (*X*^2^=4.005), the incidence of wound infection in group A was significantly lower than that in group B. The suspension bed is especially suitable for clinical treatment of patients with extensive burns.

## 2. Methods

### 2.1. Data Source

There were 40 patients, including 30 males and 10 females, with extensive burns in a department of burn and plastic surgery, aged 20–45 years, with an average age of 32.2 years. Causes of burns were as follows: 14 cases of gas, 12 cases of flame, 4 cases of hot cement, 4 cases of molten iron, 4 cases of boiled water, and 2 cases of electric shock, with all of the burn areas being above 80% of burn depth II-III, with the average burn area of III of (30 ± 15)%. There were 6 cases with mild inhalation injury, 4 cases with moderate inhalation injury, 12 cases with shock, 2 cases with ocular trauma, 2 cases with tension pneumothorax, 2 cases with renal failure, and 4 cases with stress ulcer bleeding. After admission, 4 cases underwent tracheotomy, 1 case underwent hemodialysis, and 2 cases underwent indwelling gastric tube placement, randomly divided into group A (20 cases) and group B (20 cases). There was no significant difference in gender, age, etiology, and condition between the two groups, but it was comparable [12]. The details are shown in Table 1.

### 2.2. Nursing Method

#### 2.2.1. The Preparation before Lying on the Suspended Bed

Indoor environment preparation: the suspended bed should be placed in a single room, and the indoor room should be provided with air conditioning screens. Room temperature meters and other instruments were placed to minimize unnecessary personnel flow and keep indoor air clean. Room temperature was required to be kept at 22–25°C to reduce dust. Preparation of the suspended bed unit: after receiving the notice that the patient needs to lie on the suspended bed, the suspended bed should be started early to allow the bed to slowly heat up. The setting of the bed temperature should be based on the principle that patients can tolerate and feel comfortable, generally between 30 and 36°C and between 30 and32°C in summer. It can be set at 34–36°C in winter. In addition, because the filter sheet is expensive, most burn wounds are treated with Piwei iodine yarn for external application and semiexposure. Frequent washing will easily lead to damage of the filter sheet. Therefore, in order to prevent the filter sheet from being too dirty, a layer of sterilized sheets should be laid on the filter sheet, and no other sundries should be placed on the bed [13].

#### 2.2.2. Health Education of Suspension Bed Treatment

Because of the special structure of the suspended bed, the floating bed surface, and the patient's ignorance of the mechanism of the bed and its advantages in wound treatment, the patient is in fear or doubt. In addition, the high cost of using the bed also makes the patients full of contradictions, so we should patiently explain the following to the patients before they lie on the suspended bed. The mechanism and therapeutic effect of the suspended bed are briefly introduced. The suspended bed is an air-flowing therapeutic unit filled with tiny silica sand in its container. Similar to beach, filtered air is sent to silica sand by using an air compressor, which makes the silica sand become fluid and creates a buoyancy in the rolling of silica sand. This buoyancy can minimize the pressure on the wound surface, thereby reducing the chance of infection and scab dissolution. Because of the buoyancy, there is no need to turn over before the wound dissolves the scab, which reduces the discomfort of patients on turning on the common bed (especially in the prone position) and the workload of nurses [14]. This paper briefly introduces the structure of the bed, except that the container is filled with silica sand and a breathable filter sheet is fixed on the surface of the bed with plastic modeling [15]. There is also a set of automatic water cooling, air cooling, and heat generating systems in the bed to keep the bed temperature unchanged. Because the dry and hot air blow to the wound continuously, they may not get used to it when they just lie down, but the continuous pouring of hot air and the flow of silica sand can form a warm microwave vibration effect, which can relieve the pain of the wound.

The adjustment of the infusion volume has to be paid attention to. When the patient is placed in dry and hot air, the loss of water due to evaporation increases, which is estimated to be 2 times higher than the normal loss of water. It easily causes dehydration, hypernatremia, hyperkalemia, and other electrolyte disorders. In the clinical application of 20 patients with extensive burns, 3 patients developed hypernatremia, and 2 of them were corrected in time by adjusting the fluid volume. Therefore, patients lying in suspended beds should be supplemented with more fluids. Especially for patients in the shock stage, we should closely observe the changes in urine volume, monitor the plasma electrolyte, accurately record vital signs and 24-hour urine volume, and prevent dehydration and electrolyte disturbance. The burn patients who pay attention to disinfection and isolation are all severe burn patients treated with exposure or semiexposure; therefore, it is necessary to do a good job of protective isolation. In addition to the routine disinfection and isolation work, such as ultraviolet disinfection, ventilation by opening doors and windows, mopping the floor with chlorine-containing disinfectant water, and wiping the bedside table, aseptic operation should be strictly carried out during nursing operation, and all articles used in the bed should be disinfected materials. If the patient has diarrhea, the sterilized burn gauze should be put under the buttocks and wrapped around the wounds of both thighs, minimizing the contamination to the wound surface.

### 2.3. Methods

Group A was admitted to the hospital; that is, they were made to lie on the suspended bed. Four people carried the patient; that is, first, a clean sheet was placed under the patient, and four people were located near the head and feet and near the waist and hips on the left and right sides [16]. Together, they lifted the sheet and placed the patient in the supine position on the suspended bed steadily. The veil pad was replaced once or twice a day, without turning over for three weeks, using the buoyancy of the bed to flip the patient. The rehydration is calculated as 2–4 ml/(kg h); blood electrolytes were checked once in 12–3 days. In order to prevent dehydration and hypernatremia and hyperkalemia of patients, the room temperature was kept at 20–24°C to reduce the heat dissipation load of the suspended bed. In group B, the bed was turned over immediately after admission. The bed was turned over once every 4 hours to prevent the back wound from being compressed through the alternate exposure of the front and back wounds of the trunk. After each turn, the wet gauze pad of the wound needs to be replaced, and the height of the shelf of the patient's head and limbs should be readjusted. In the prone position, the changes of respiration and heart rate should be closely observed to prevent asphyxia. Nurses should bend over or squat down to feed the patients with conventional rehydration [17]. After 3 weeks, the patients in both groups laid on the bed, and according to the standard of nursing evaluation of conventional treatment, observation of the dry scab of the wound at 16 hours after treatment and wiping of the scab skin of the wound with sterile gauze were performed, and the scab will be dry if there is no seepage. Fourteen days after treatment, the wound healing was observed. The third wound healed with a red, tender, and smooth new epithelium exposed after necrotic epidermis shedding, and the second wound healed after escharectomy and skin grafting or skin flap transplantation. On the 10th day after injury, bacterial culture was carried out to confirm whether there was invasive infection [18].

## 3. Results and Analysis

### 3.1. The Curative Effect and Nursing Time of Patients in the Two Groups

**Figure 1:**
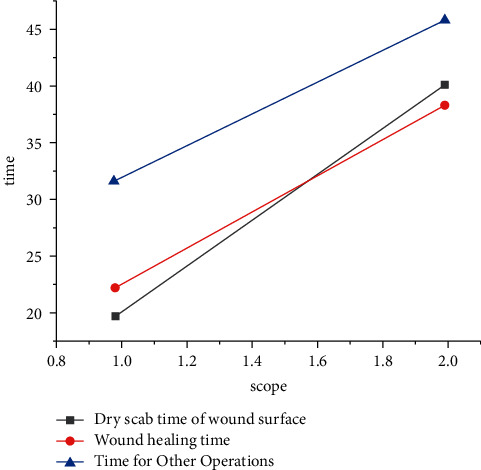
Curative effect and nursing time in group A.

**Figure 2:**
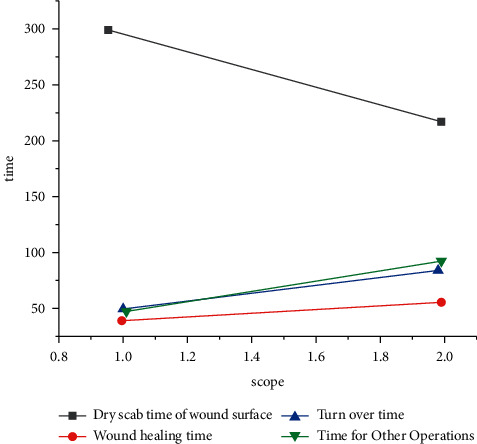
Curative effect and nursing time consumption in group B.

As shown in Figures 1 and 2, on the 10th day after injury, 4 cases (20%) were positive in group A and 8 cases (40%) were positive in group B. Comparison was made between the two groups, *X*^2^=4.005. The incidence of wound infection in group A was significantly lower than that in group B. The comparison of the curative effect and the nursing time between the two groups is shown in Table 2.

### 3.2. Mechanism of Action of the Suspended Bed

The suspended bed is mainly composed of an elliptical large container, breathable filter compressor, heat radiator of heat generator, air filter, computer control panel of water inlet and drainage pipe of water filter, foot switch, and other components. The large container is 223 cm in length, 91 cm in width, and 90 cm in height and contains 681.8 kg of 50–1501 m fine silica sand. Two detachable plastic shapes are arranged around the container for sealing and fixing the filter sheet. The computer control panel for preventing silica sand from overflowing with air is used to set the bed temperature, suspension type, and duration. The foot switch is used to suspend suspension at any time during nursing operation. When the existing bed temperature is lower than the set bed temperature, the heat generator is started immediately, and the tap water filtered by the water filter is sucked into the bed for circulating heat generation to raise the bed temperature. When the existing bed temperature is higher than the set room temperature, the radiator is started, the water carrying the heat in the bed is discharged out of the bed through the drainage pipe to achieve the effect of water cooling and heat dissipation, and the bed temperature is kept constant. In the suspended state, the air compressor transmits the air filtered by the filter into the silica sand so that the silica sand becomes fluid, and the silica sand moves to form buoyancy, which effectively prevents the back wound surface from being pressed. At the same time, dry hot air can be poured onto the wound surface through the filter sheet. A therapeutic environment in which dry and hot air continuously surrounds the patient is formed [19].

### 3.3. Suspension Bed Can Accelerate Wound Healing and Reduce Infection Rate

In the process of using the roll-over bed, some patients with extensive burns and organ injuries can or cannot tolerate prone, so the number and time of prone cannot be guaranteed, resulting in compression of the annular wounds of the trunk and limbs. The suspension bed not only does not need to turn over, which is convenient for rescue, but also can continuously surround the patient with dry and hot air. Table 2 shows that the time of scab drying and wound healing in group A is significantly less than that in group B. The suspension bed has a unique function of controlling microbial infection. When the patient's exudate is absorbed by silica sand, it becomes agglomerate due to the increase in weight and sinks into the screen at the bottom of the bed to prevent contact with the patient. Even the microorganisms remaining on the filter sheet can be dried out and lose their vitality due to the upward movement of air. Relevant research shows that most of the bacteria submerged in silica sand lose their vitality after using the suspension bed for 24 hours, and bacteria that have not been inactivated will be inactivated after staying in the sand for only 48 hours. This is due to the increase of pH value caused by the precipitation of sodium ions when the patient's exudate comes into contact with silica sand. This is an important reason for the decrease in infection. Due to the dry and hot air environment, the moisture of patients is evaporated in large quantities, which easily causes dehydration, leading to hypernatremia and hyperkalemia. Patients should be given plenty of water and increased intravenous rehydration, and their blood electrolytes should be checked regularly. Hypernatremia occurred in 1 case, and dehydration was corrected after the abovementioned treatment [20].

### 3.4. Relieving the Patient's Wound Pain

Because of the small bed surface, the patient's posture change is limited. After turning over, the wet gauze pad should be removed from the wound surface. Every time the patient turns over, he/she needs to endure severe pain. Because the suspension bed does not need the patient to turn over and the suspension bed is 1/3 wider than the turning bed, the buoyancy can minimize the wound friction when the patient changes posture. In addition, the movement of silica sand and the continuous pouring of hot air on the wound surface of patients can achieve physiotherapy effect and paralyze the nerve endings in the compressed area. Therefore, the suspension bed can alleviate the wound pain of burn patients.

### 3.5. Reduction of Nursing Difficulties

Two nurses should cooperate to turn over the patient once every 4 h to prevent the wound from being pressed, while the floating bed disperses the patient's weight and exerts even pressure on any part of the body because of the buoyancy. Thus, the wound can be effectively prevented from being pressed without turning over, and the buoyancy can be used to make it easier to change the body position during life care and dressing change, which greatly saves nursing manpower and material resources and shortens working hours. However, the suspended bed is expensive, so patients should have certain economic bearing capacity.

### 3.6. Treatment and Disinfection of the Suspended Bed after Use

After use, the patient thoroughly wipes and disinfects the bed unit with a disinfectant. The filter sheet is removed and rinsed with soapy water. The stain on the filter sheet cannot be removed after cleaning twice, but can be cleaned with an oxidizing disinfectant. If a patient is lying on the suspension bed and the filter sheet is polluted, the suspension bed can be suspended, and the stain is wiped with a towel soaked in hot soap first, and then, the soap is wiped clean with a wet towel. Finally, the removal of water is carried out with a dry towel, and the filter sheet quickly dries under the hot air circulation. Every six months, removal of sand grains, foreign matters, crystals, etc. from the screen should be performed.

## 4. Conclusions

In this paper, we explore the nursing methods of applying multifunctional intelligent suspension treatment beds in patients with extensive burns. The advantages of the suspension bed include the ability to reduce pressure on the wound surface to a minimum, thus reducing the risk of infection and scab dissolution. Because of the buoyancy, there is no need to turn over before the scab on the wound surface dissolves, which will reduce the discomfort of patients' common bed (especially in the prone position) and the workload of nurses. Suspension beds are especially suitable for the clinical treatment of patients with extensive burns.

## Figures and Tables

**Table 1:** Basic information.

	Number of cases	Gas	Flame	Hot cement	Molten iron	Boiling water	Electric shock
A	20	4	5	2	4	3	2
B	20	10	7	2	0	1	0

**Table 2:** Comparison of the curative effect and nursing time between the two groups.

Therapeutic effect	A	B
Time of wound scab drying	28.92 ± 9.02	256 ± 44.58
Wound healing time	31.45 ± 6.64	41.45 ± 6.69
Time taken for turning over	0	58.75 ± 5.33
Time taken for other operations	39.75 ± 6.53	59.76 ± 7.62

## Data Availability

The data used to support the findings of this study are available from the corresponding author upon request.
